# Electrospun Cellulose Nanocrystals/Chitosan/Polyvinyl Alcohol Nanofibrous Films and their Exploration to Metal Ions Adsorption

**DOI:** 10.3390/polym10101046

**Published:** 2018-09-20

**Authors:** Dong Wang, Wanli Cheng, Yiying Yue, Lihui Xuan, Xiaohui Ni, Guangping Han

**Affiliations:** 1 Key Laboratory of Bio-based Material Science and Technology (Ministry of Education), Northeast Forestry University, 26 Hexing Road, Harbin 150040, China; sdzcwangdong@nefu.edu.cn (D.W.); nefucwl@nefu.edu.cn (W.C.); leeh91@163.com (L.X.); wnixiaohui@163.com (X.N.); 2 College of Biology and the Environment, Nanjing Forestry University, Nanjing 210037, China; yue@njfu.edu.cn

**Keywords:** electrospining, chitosan, cellulose nanocrystals, nanofibrous films, metal adsorption

## Abstract

Cellulose nanocrystals/chitosan/polyvinyl alcohol (CNC/CS/PVA) composite nanofibrous films were prepared while using an electrospinning technique and successfully thiol-functionalized. Then, the modified films were used for the sorption-desorption of Cu(II) and Pb(II) ions. Subsequently, the adsorption capacity of the films was investigated by changing the CNC loading level, solution pH, and adsorption time. Results showed that the adsorption of metal ions by the films was the best with CNC loading level of 5 wt %, pH of 6, and adsorption time of 4 h. The adsorption behavior of the films was agreed with the Freundlich model. The adsorption equation of metal ions could be described while using a pseudo-second order model. Based on the Langmuir model, the maximum adsorption capacities of Cu(II) and Pb(II) ions were estimated to be 484.06 and 323.49 mg/g, respectively. The Cu(II) and Pb(II) ions adsorption efficiencies of the films after 4 adsorption-desorption cycles were 90.58% and 90.21%, respectively. This study may provide a feasible approach for the application of functional CNC/CS/PVA nanofibrous films in the treatment of water.

## 1. Introduction

Heavy metals have been widely applied in industries, such as electroplating, metal processing, and leather manufacturing. It is inevitable that metal-contaminated materials have been released into our environment, posing a great threat to environmental safety and human health [1,2]. Heavy metals can cause serious ailments, such as lung insufficiency, liver damage, and destruction of nervous systems of young children [3]. The effective removal of toxic metal ions from contaminated water has been extensively studied.

Adsorption (low-cost, easy recovery of metals, and possible reuse of the adsorbent) is regarded as one of the most effective and cost-efficient approaches for the removal of toxic metal ions from water. A variety of adsorbents, such as activated carbons, natural zeolites, biosorbents, and chelating materials [4,5,6] have been employed for the adsorption of metal ions. Lignin, cellulose, and chitin are the most well-known natural polymers, put directly into the polluted solution generally [7]. They can be activated or modified to improve their ability to remove heavy metals. For example, chitosan (CS), which is the deacetylation product of chitin, can remove Cadmium, Cobalt, Copper, or Lead [8,9], because of the rich amino, hydroxyl, and carbonyl groups of the molecular chain [7]. Surface area is another important factor that affects the adsorption capacity, efficiency, and durability of the materials [10].

Many studies have also shown that the adsorption performance of the membrane is better than that of microspheres and powders [11,12,13,14]. Electrospinning is a technique that is used for the preparation of ultralong one-dimensional fiber materials with diameters in the nanometer scale [15]. The remarkable characteristics of electrospun CS materials are high surface area per unit mass, spongeous nature with small pores, and wet absorption, which may contribute to fast and high adsorption, owing to exposure of the CS nanofiber’s functional groups to the metal ions [16,17]. Owing to the high crystallinity of CS, the number of solvents that can be used for electrospinning is low, which is a limitation of electrospinning CS nanofibers [18].

Poly(vinyl alcohol), PVA, is a water-soluble polymer that is commonly used to improve the spinnability of CS [19,20,21,22]. However, the interfacial compatibility of electrospun CS/PVA composite nanofibers is poor, and the loose parts of the nanofibers are easily detached in an aqueous solution, resulting in the weak mechanical and reusability properties [23]. For environmental considerations, the materials used as reinforcement agents in composite nanofibrous films should be renewable. Among such materials that are readily available, CNC have attracted great interest owing to its renewability, biodegradability, and spectacular mechanical properties [24,25]. Incorporating CNC into electrospinning of different polymers can improve the mechanical and thermal properties of nanofibers [26]. Xu introduced CNC into the CS/PVA matrix to improve its insufficient mechanical properties. The results showed that when the CNC mass fraction was 5 wt %, the mechanical properties of the nanofibers significantly improved [19]. However, the reactive sites of composite nanomaterials were limited [27]. The introduction of functional groups, such as carboxyl, amino, and thiol groups [28,29] onto the surface of electrospun fibers could further improve their utilization as adsorbents and have shown excellent adsorption capacity in removing heavy metals [30].

In this work, electrospun materials that are based on a CS/PVA matrix reinforced with CNC were prepared while using an electrospinning technique and then the composite nanofibrous films were further functionalized with thiol groups using thioglycolic acid. Characterization of the obtained composite nanofibrous films was analyzed by scanning electron microscopy (SEM) and fourier transform infrared spectroscopy (FTIR). In addition, the microstructure of the nanofibrous films in different acid-base solutions was analyzed, and the optimum CNC loading and pH of the films for metal ions were investigated. Finally, the adsorption behavior and kinetics of the electrospun CNC/CS/PVA-SH composite nanofibrous films towards selected metal ions (M(II), Cu(II), or Pb(II)) were demonstrated while using atomic absorption spectroscopy (AAS). The durability of the material was evaluated by repetitive adsorption-desorption experiments. This study was aimed to provide theoretical support for the fabrication of electrospun composite nanofibrous films, and further provide a feasible approach for the application of electrospun composite nanofibrous films in the water environment field.

## 2. Materials and Methods

### 2.1. Materials

PVA (Degree of alcoholysis 98 ~ 99%, M_w_ = 220,000), CS (Degree of deacetylation 95%, M_w_ = 200,000), thioglycolic acid, copper chloride, and lead chloride were purchased from Aladdin (Shanghai, China). Microcrystalline cellulose (MCC, moisture content = 75%, Daicel Co., Ltd., Tokyo, Japan), sulfuric acid (H_2_SO_4_), acetic acid (CH_3_COOH), tetrahydrofuran (THF), sodium hydroxide (NaOH), and hydrochloric acid (HCl) were purchased from Kermel Co., Ltd. (Tianjin, China). Deionized water was used throughout the study. All of those agents were used as received without further purification.

### 2.2. Preparation of CNC

The preparation of CNC by H_2_SO_4_ hydrolysis is as follows. 40 g of MCC was mixed with 4.7 g of water in flask, before dropping 65.3 g of 98 wt % H_2_SO_4_ by a constant pressure dripping funnel under vigorous magnetic stirring, followed by placing the flask in an ice-water bath (low temperature pre-reaction). After 0.5 h (process of drop H_2_SO_4_), the flask was quickly transferred to 45 °C water bath device for 1 h. The hydrolysis process was stopped by adding additional water. CNC were isolated from the obtained mixture by combining centrifugation (10,000 rpm and 10 min) and ultrasonic (400 w, 15 min) and dialysis (7 days) at ambient temperature. Then, the CNC were dried while using a freeze dryer (Scientz-10N, XinZhi Co., Ltd., Ningbo, China) before being stored in a cold environment for use. The CNC obtained by direct H_2_SO_4_ hydrolysis (without low temperature pre-reaction process) were used as a comparison.

### 2.3. Electrospinning

The spinning solution used in this work was prepared as described below. The CS powder was dissolved in a solution of acetic acid and water (90:10 vol %). An aqueous solution of PVA was prepared at 90 °C for 2 h. The neat CS and PVA mass fraction (wt %) was kept constant at 3 wt % and 12 wt %, respectively. A mixture of the CS and PVA spinning solution was prepared at a volume ratio of 60:40 under magnetic stirring at room temperature for 10 h. The CNC loading percentages in relation to the CS/PVA were 0, 5, 10, and 20 wt %.

Electrospinning was carried out by using a horizontal setup (Yong Kang Le Ye Co., Ltd., Beijing, China) that included a 10 mL syringe (containing an 18 G needle with an inner diameter of 0.9 mm) connected to a syringe pump. The syringe pump was used to supply a steady spinning rate (0.4 mL/h) to the tip of the needle. A high-voltage power supply was used to apply a potential of 17 kV to the syringe needle. A roller collector device (covered with a layer of aluminum foil) with −3 kV was rotated at a speed of 80 rpm and then placed at a distance of 18 cm from the needle tip. The relative humidity and temperature were constantly monitored and maintained at 22% and 25 °C, respectively. The samples were designated as *x*CNC/CS/PVA, in which *x* (wt %) was the CNC loading level.

### 2.4. Modification of xCNC/CS/PVA with Thioglycolic Acid

A certain amount of composite nanofibrous films were placed in a solution of thioglycolic acid and THF at a volume ratio of 1:1, with 0.2 wt % H_2_SO_4_ loading [31]. The reaction mixture was gently stirred at room temperature for 3 days The thiol-functionalized nanofibrous films were obtained, which were named as *x*CNC/CS/PVA-SH. The nanofibrous films were first washed with deionized water until the solute was neutral. The nanofibrous films were dried at −56 °C for 24 h while using a freeze dryer and sealed in a plastic bag for storage at room temperature.

### 2.5. The Stability of xCNC/CS/PVA-SH Nanofibrous Films

To investigate the stability of the *x*CNC/CS/PVA-SH composite nanofibrous films under different acidic and alkali conditions, the pH of the deionized water was adjusted to 2.0 ~ 10.0 using 0.01 mol/L HCl or 0.01 mol/L NaOH. The immersion time and temperature were maintained at 24 h and 25 °C, respectively.

### 2.6. Adsorption of the Metalions

In this work, the concentration of the metal ion solution was maintained at 60 mg/L and the volume that was used for the experiments was 100 mL. Approximately 50 mg of CNC/CS/PVA-SH composite nanofibrous films were used as adsorbent material. All of the samples were analyzed at room temperature (25 °C) and analyzed in three parallel experiments.

To investigate whether the incorporation of CNC into the CS/PVA composite nanofibrous films improved the adsorption capacity for M(II). The *x*CNC/CS/PVA-SH films were added to a M(II) solution at pH 6 for 24 h. The adsorption capacity of films was investigated by changing the solution pH, and adsorption time. The pH was set to 4.0 ~ 10.0, The residual M(II) solution concentration was detected after 24 h, and a 5 μL of the M(II) solution supernatant was taken at pH 6 for 0, 1, 2, 4, 8, 12, and 24 h. To investigate the effect of metal ion concentration on the adsorption capacity of films, the metal ion concentration was varied from 50 to 800 mg/L, and the pH value was adjusted to 6. To further investigate the repetitive adsorption of composite nanofibrous films for M(II), the films were evaluated by repetitive adsorption-desorption experiments. The desorption of M(II) was performed in 4 mol/L HCl aqueous solution (20 mL) for 12 h. The nanofibrous films that were used for desorption were washed with deionized water to neutral and then freeze-dried for 2 d and the sample was weighed before it was used in the next adsorption-desorption investigation.

### 2.7. Characterization of xCNC/CS/PVA Composite Nanofibrous Films

The micromorphology and diameter size of the obtained CNC were characterized by transmission electron microscopy (TEM, Hitachi-7650, Electron Optics Labotatory Co., Tokyo, Japan) and Nanomeasure software 1.2. For the analysis, the aqueous CNC suspensions were diluted to 0.05 to 0.1 wt %. The crystallinity of CNC was determined via X-ray diffraction (XRD, D/max-2200VPC, Rigaku Co., Ltd., Tokyo, Japan). The equation *%Cr*_I_ = 100 × *A*c/*A*a was used to calculate the *Cr*_I_, where *A*c was the area of the crystalline region and *A*a was the area of the entire diffraction peak. Optical microscopy images of the spinning solution were created while using a Nikon (Ci-E/Ci-L/Ci-S, Electron Optics Labotatory Co., Tokyo, Japan) imaging system. Properties (viscosity, surface tension, and conductivity) of as-spun solutions were measured using a digital rotational viscometer (SNB-1, Heng Ping Co., Ltd., Shanghai, China), surface tension meter (JK 98B, Zhong Chen Co., Shanghai, China), and conductivity meter (DDSJ-318, Lei Ci Co., Ltd., Shanghai, China), respectively. The microstructure of the nanofibrous films was observed while using SEM (QUANTA-200, FEI Co., Hillsboro, OR, USA). The films were coated with a layer of gold palladium, and element distribution was obtained by SEM-mapping. The characteristic absorption peaks of the electrospun nanofibrous films were examined by FTIR (NICOLET 6700, Thermo Fisher Scientific Co., Ltd., Agawam, MA, USA). The zeta potential (*ζ*) of the sample in different pH solutions were analyed using a Laser particle size analyzer (Zetapals, Bruker Co., Ltd., Karlsruhe, Germany) and the values were tested three times to ensure the reliability. The concentration of the M(II) remaining in the solution was analyzed using AAS (NovAA400P, Bruker Co., Ltd., Karlsruhe, Germany).

## 3. Results and Discussion

### 3.1. Characterization of CNC

**Figure 1:**
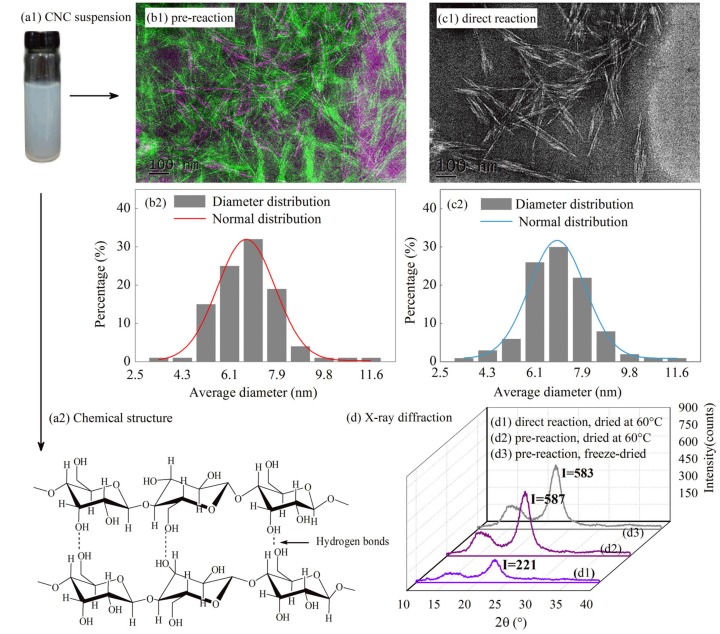
The cellulose nanocrystals (CNC) obtained by acid hydrolysis: (**a1**,**a2**) The CNC suspension and chemical structure; (**b1**,**b2**) and (**c1**,**c2**) transmission electron microscopy (TEM) images and the size distribution; and, (**d**) X-ray diffraction (XRD) of CNC obtained by different preparation and drying conditions.

The TEM images and size distribution of short rod-like CNC obtained by acid hydrolysis are shown in Figure 1. The results showed that the pre-reaction process resulted in a more uniform distribution of CNC diameters, with an average diameter of 6.8 ± 4 nm, an average length of 96 ± 32 nm, and average aspect ratio of 14.10, while the average diameter and length of CNC that were prepared by direct reaction were 6.2 ± 6 nm and 90 ± 35 nm, respectively, and average aspect ratio was 14.51. Huan has reported that the diameter and length of CNC was 9 ± 3 nm and 90 ± 28 nm and average aspect ratio of 10 by direct acid hydrolysis [32]. This result can be explained by the low temperature (ice bath environment), which makes the initial reaction process more stable.

The crystal properties of the CNC obtained by three different processing methods are shown in Figure 1d. The three kinds of CNC had characteristic peaks at 2θ = 22.8° and 2θ = 15.2°, corresponding to the strong peak of cellulose *I*_β_ (200) and the weak cellulose *I*_β_ crystal surface peak [33], respectively. The crystallinities (Figure 1d1–d3) calculated by MDI Jade 6.5 were 58%, 72%, and 69%, respectively. The acid hydrolysis process (whether the ice bath process was used) had a significant influence on the crystallinity of the obtained CNC, whereas the drying conditions had no effect on the crystallinity of the obtained CNC.

The main reason for this difference was that in the pre-reaction stage, the molecular chains of MCC were frozen at low temperature and it was difficult for H_2_SO_4_ to enter the molecular chain structure of MCC. Therefore, the reaction was limited to the surface of the MCC molecular chain. In the constant temperature reaction stage, the molecular chains of MCC were completely open in the acid solution and H_2_SO_4_ can enter the non-crystalline region and the MCC molecular chain underwent a hydrolysis reaction. Therefore, the CNC that were prepared by pre-reaction and freeze-dried process were used as the raw material for subsequent experiments.

### 3.2. Surface Morphology of the xCNC/CS/PVA Composite Nanofibrous Films

**Figure 2:**
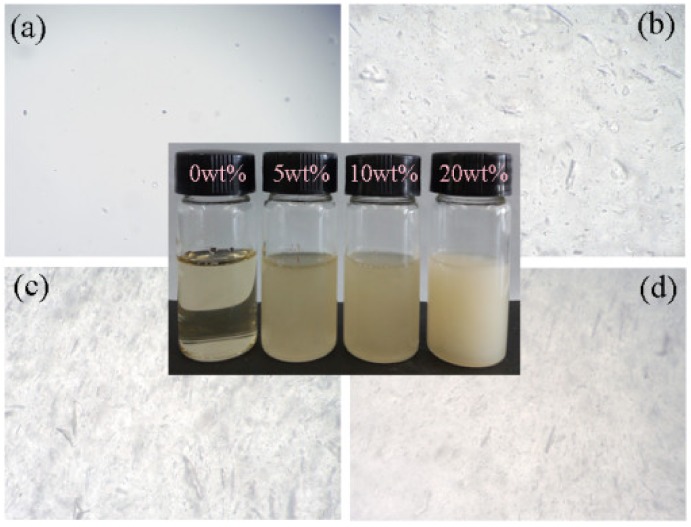
Optical microscopy images of mixed solution with different CNC loading level: (**a**) 0 wt %; (**b**) 5 wt %; (**c**) 10 wt %; and (**d**) 20 wt %. The inset images represent the photograph of spinning solutions with various CNC contents.

**Figure 3:**
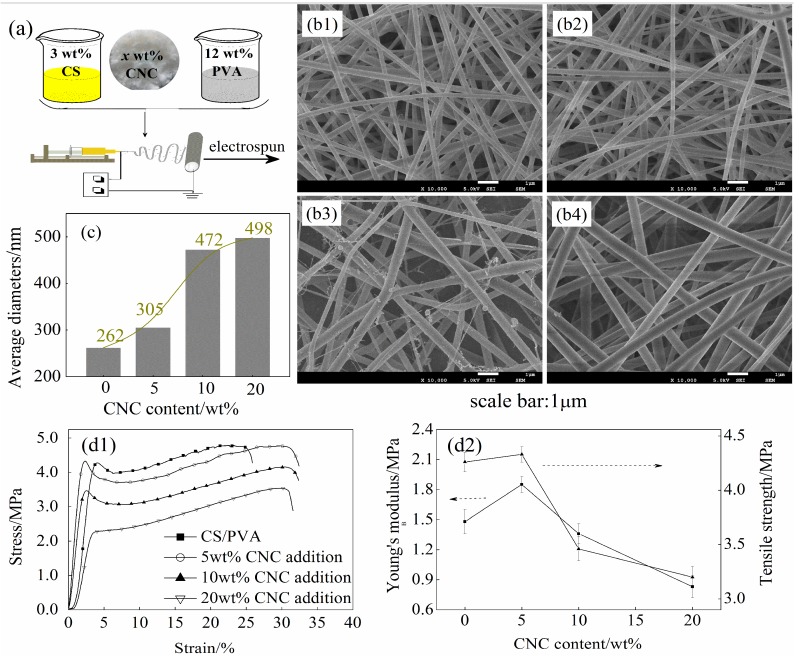
Electrospun cellulose nanocrystals/chitosan/polyvinyl alcohol (CNC/CS/PVA) composite nanofibrous films (**a**) Schematic diagram of preparation process; (**b1**–**b4**) Surface morphology of nanofibrous films with varying CNC loading levels: (**b1**) 0 wt %, (**b2**) 5 wt %, (**b3**) 10 wt %, (**b4**) 20 wt %; (**c**) Average diameters of nanofibrous films; and, (**d1**,**d2**) Stress-strain curve (**d1**) and tensile properties (**d2**) of nanofibrous films.

Figure 2 shows optical microscopy images of mixed spinning solution. It can also be seen that CS/PVA solution was semitransparent, while the spinning solution turned to be increasingly turbid with the increase of CNC loading level. In Figure 3a, the *x*CNC/CS/PVA composite nanofibrous films can be prepared while using an electrospinning technique. The surface morphology and calculated average diameters of electrospun *x*CNC/CS/PVA nanofibrous films are shown in Figure 3b,c. With the increase of CNC loading level, the surface of the fibers changed from a smooth state to a rough one. When 5 wt % of CNC was added, the diameter of nanofibers was uniform and regular. When 20 wt % of CNC was added, the diameter became un-uniform; nanofibers with a small diameter and spherical substances attached to the single nanofiber surface were observed at the same time. The main contributor was the higher polarity of the CNC, which was not evenly distributed in the CS/PVA matrix, making the jet unstable during the electrospinning process and resulting in the splitting of the jet. The diameter of nanofibers increased with increasing CNC loading level. The CS was a polycation with a positive charge and the CNC had negative charges. During electrospinning, the CS molecules preferred to move out from the effect of the electrical field and electrostatic repulsion [34], resulting in increased nanofiber diameter. Zhang also observed this phenomenon when using electrospinning technology to prepare single-spun core-shell structure polyethylene oxide/CS composite nanofibrous films [35].

As shown in Figure 3d, the Young’s modulus (*E*) and tensile strength (*σ*) of *x*CNC/CS/PVA nanofibrous films first enhanced and then weakened with the increase of CNC content. When the content of CNC was 5 wt %, the nanofibrous films exhibited the best mechanical properties. When compared with CS/PVA nanofibrous films, *E* and *σ* of the nanofibrous films with CNC incorporation increased by 16.4% and 25.0%, respectively. This was likely attributed to the fact that low added amount of CNC can be evenly distributed in the CS/PVA matrix (Figure 2b), leading to closer hydrogen bonding between the polymer molecules. At this time, the cooperative interaction between the CNC and the CS/PVA matrix was better. In addition, the viscosity, surface tension, and conductivity of the spinning solutions play a decisive role in the morphology of nanofibrous films [36]. Thereby, it is possible to influence the mechanical properties of the nanofibrous films. As shown in Table 1, after adding CNC, the viscosity became smaller and the surface tension became larger. The conductivity increased with the increase of CNC, mainly because the addition of CNC made the solution charge density increase. Under the combined action of the above factors, the mechanical properties of nanofibrous films have changed to varying degrees with the increase of CNC content.

After the addition of CNC, the tensile properties of the nanofibers were deteriorated due to the poor compatibility between the components of the CNC/CS/PVA nanofibrous films. Furthermore, the blend polymer can improve the mechanical properties of composite materials [37]. However, the rough nanofiber surface, uneven diameter distribution, and disorderly arrangement may act as “defects” that can negatively affect the mechanical performance of nanofibrous films by forming cracks prematurely during the tensile test [38].

### 3.3. FTIR of Electrospun xCNC/CS/PVA-SH Nanofibrous Films

**Figure 4:**
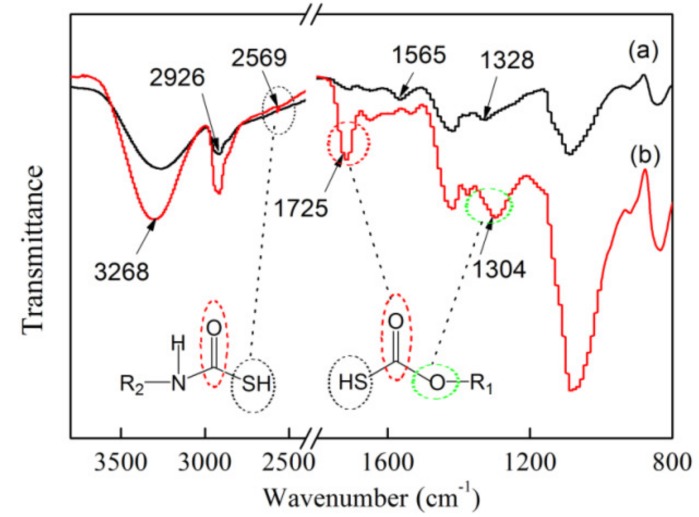
Fourier transform infrared spectroscopy (FTIR) of the CS/PVA/CNC composite nanofibrous films (**a**) before, (**b**) after modification.

The electrospun *x*CNC/CS/PVA composite nanofibrous films that were treated with thioglycolic acid were subjected to analysis by FTIR. The absorption band was not only related to the chemical composition of the unit, the connection between the monomers, and the molecular content, but also the size of the particle. As shown in Figure 4a, the broad band between 3350 and 3250 cm^−1^ was attributed to the –OH and N–H of the polysaccharide molecule. 2920 cm^−1^ was the stretching vibration peak of –CH_2_ and 1558 ~ 1648 cm^−1^ was the deformation vibration peak of –NH_2_, indicating the presence of the amino group with high adsorption properties. The acetamide groups (–CH_3_(CO)NH_2_) and C–CH_3_ deformation vibration peak of CS appeared at approximately 1325 cm^−1^ [39]. Figure 4b. shows the FTIR spectrum of the nanofibrous films after modification. A strong and narrow peak appeared at approximately 1725 cm^−1^, which was mainly due to the condensation reaction of –OH and –COOH and the generation of an ester carbonyl (C=O). Support for this explanation was provided by changes in the peak structure of the band observed at 1325 cm^−1^, which was the characteristic peak of C–O–C. The weak characteristic absorption band at 2573 cm^−1^ was attributed to –SH [40], which were exposed on the surface of the materials. The difference in the shape of the characteristic absorption bands in Figure 3a,b indicated the successful thiol-functionalization of the *x*CNC/CS/PVA nanofibrous films.

### 3.4. Surface Morphology of the CNC/CS/PVA-SH Composite Nanofibrous Films

**Figure 5:**
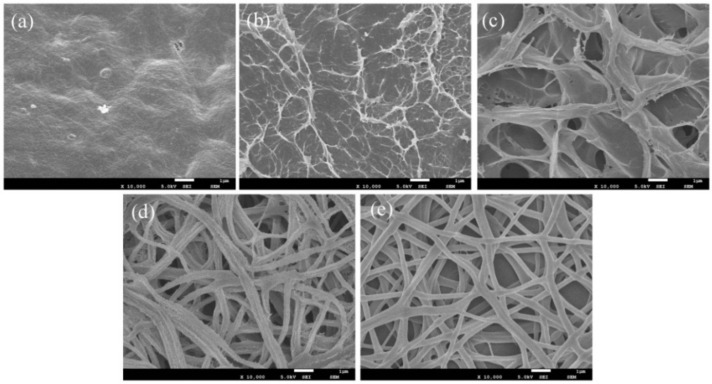
SEM images of electrospun 5CNC/CS/PVA-SH composite nanofibrous films after treatment with solution at (**a**) pH 2, (**b**) pH 4, (**c**) pH 6, (**d**) pH 8, and (**e**) pH 10 (scale bar: 1 µm).

Figure 5 shows the effect of pH on the surface morphology of 5CNC/CS/PVA-SH composite nanofibrous films. The CS is soluble in acid solution and the PVA is soluble in water, allowing the nanofibrous films to self-contract after immersion in aqueous solution as a result of surface tension. As shown in Figure 2b and Figure 5a, it is quite hard for the nanofibrous films to keep their nanofiber morphology in aqueous solutions. The microstructure of the nanofibrous films was severely destroyed and the surface was rough in strong acidic medium. The nanofibers tend to merge together into a monolithic film in a solution of pH = 6, and its surface area decreased substantially.

With a decrease in the acidity of the solution, the surface of the nanofibrous films began to exhibit a series of flocculent protrusions; then the membrane surface gradually became irregular and cross-linked with an extremely rough layered structure. When the pH of the solution was close to neutral, this layered structure was reduced and the linear structure began to appear. The reason for this phenomenon can be attributed to swelling of the CS component and the protonated reaction in acidic media [41], and a part of CS gathered on the surface of the films in the form of a gel. When the nanofibrous films were treated with a weak alkaline medium, the nanofibrous films were linear with a cross-linked structure, and the surface of the linear structure appeared as a series of needle-like protrusions. As the alkalinity increased, the surface of the composite films changed into a smooth linear structure. When compared with the CNC/CS/PVA nanofibrous films in Figure 2b, more of the original structure of the material was retained at higher alkalinity (Figure 5).

The significant improvement of the nanofibrous film structure may be owing to the conversion of amino (–NH^3+^) in the nanofibrous films component to an amino group (–NH_2_) in the alkaline solution, resulting in the curing reaction of the CS matrix [16]. Therefore, the higher alkalinity, the less fiber structure is changed upon treatment with an alkaline solution. This would affect its metal adsorption behavior. However, there are many factors affecting metal adsorption, such as the pH, the activity of surface functional groups (–NH_2_, –SH, –OH, –COO–), the number of active sites, the morphology of nanofibrous, and the stability of nanofibrous during adsorption [7,42,43,44]. Therefore, it is necessary to explore the adsorption behavior of metal on the composite nanofibrous films in the presence of other factors.

### 3.5. Adsorption and Desorption of the Metalions

#### 3.5.1. Effect of Different CNC Loading Levels

**Figure 6:**
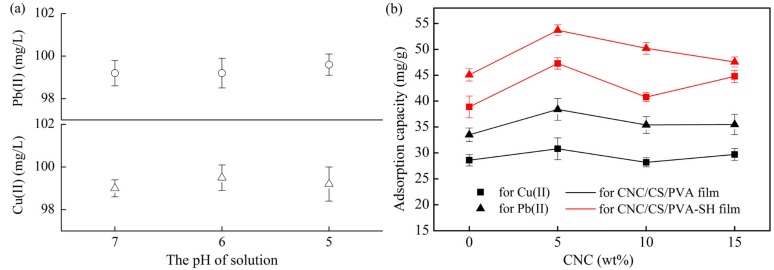
(**a**) M(II) concentration (100 mg/L) curves as a function of pH; (**b**) Adsorption capacity of *x*CNC/CS/PVA and *x*CNC/CS/PVA-SH composite nanofibrous films.

The pH of solution is an important factor for adsorption of the metal ions and it affects the precipitation of metal hydroxide. Figure 6a presents the concentration of heavy metal ions at pH 4 ~ 7. The analysis revealed that pH had little effect on hydroxide precipitation at pH 6. Previous research also showed that the metal ions exhibited different valence distribution at pH > 5.0, thus, there was no precipitation occurrence at this pH [43]. The study from Yang et al. showed that precipitations of metal ions occurred when the pH value was higher than 7.0 [42]. The adsorption capacities of the composite nanofibrous films before and after chemical modification are shown in Figure 6a,b. Regardless of the amount of CNC added, the adsorption capacities of the modified nanofibrous films for Cu(II) or Pb(II) were significantly improved. Therefore, the next studies will consider discussing the adsorption properties of the *x*CNC/CS/PVA-SH nanofibrous films.

In Figure 6b. The results showed that incorporation a small amount of CNC into the CS/PVA nanofibrous films improved the adsorption capacity for M(II). The adsorption performance for Pb(II) was better than that for Cu(II). The adsorption capacity of the composite films for M(II) reached maximum of 47.28 mg/g and 53.70 mg/g, respectively, at a CNC loading of 5 wt %. This result could be attributed to the improvement of surface structure through the introduction of CNC into the CS/PVA mixed matrix, and the polarity of the CNC surface, which was rich in hydroxyl functional groups, increasing the affinity to M(II). The adsorption capacity decreased with a further increase of the CNC content. When the CNC content was high, the high-voltage electrostatic force caused repulsion between CS and CNC, which resulted in a nonuniform microstructure, and worse adsorption properties of nanofibrous films.

#### 3.5.2. Effect of pH

**Figure 7:**
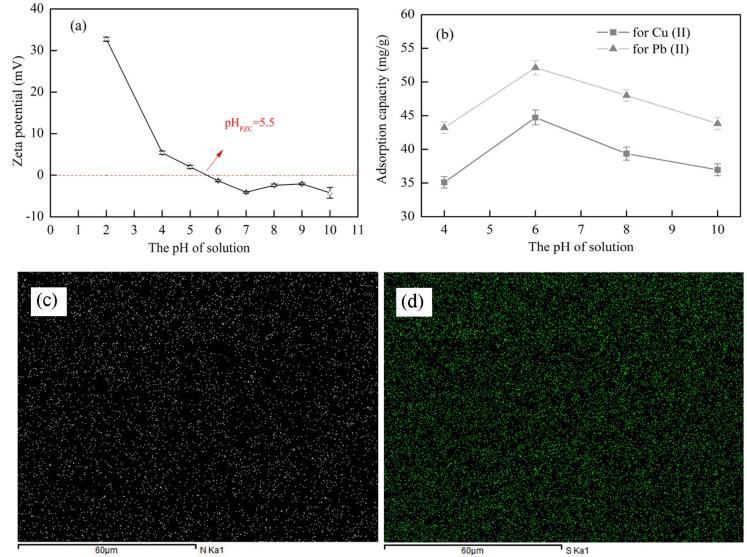
(**a**) Zeta potential curves as a function of pH; (**b**) The metal ion adsorption capacity of *x*CNC/CS/PVA-SH composite nanofibrous films at different pH; (**c**) N mapping of (**d**) S mapping images. (A colour version of this image can be viewed online.)

The pH of the solution can change the presence of pollutants and the surface charge characteristics of the adsorbent. Therefore, pH usually has a significant effect on adsorption performance. Figure 7 shows the effects of pH on the M(II) adsorption capacity of *x*CNC/CS/PVA-SH composite nanofibrous films, the distribution of N and S elements was observed by SEM-mapping. Within the test pH range, the maximum adsorption capacity of Cu(II) and Pb(II) was achieved at pH 6. The nanofibrous films swelled in the weak acid and then changed to an overlapping cross-linked layered structure, as shown in Figure 5c; and, this exposed more active sites of the nanofibrous films to the solution. Furthermore, when compared with the strong acid solution, the –NH_2_ and –SH content, with excellent metal chelating ability, was increased in weak acid solution. The adsorption capacity was the best under the combined action of both phenomena.

The effect of pH in this work was similar to that reported by Shan et al. [45]. When the pH was very low, the surface structure of the films limited the number of adsorption sites on the materials, and high concentration of H^+^ ions had a competitive adsorption with M(II) ions [42]. Furthermore, in an acid environment, the ionization state of functional groups (–SH, –OH, and –NH_2_) in nanofibrous films would change [46]. The –NH_2_ group was protonated (with positive charge), resulting in an electrostatic repulsion between the surface of films and the M(II) ions, which decreased the adsorption capacity. The adsorption capacity of films decreased when the pH value was high. This was because –NH_2_ and –OH formed NH_2_OH–, reducing the active sites that were exposed to the solution. The poor metal complexation ability of –SH under the alkaline environment can also explain this phenomenon.

Zeta potential (*ζ*) of synthesized films were measured and the results are shown in Figure 7a. It can be observed that the M(II) adsorption onto CNC/CS/PVA-SH is a function of pH and three sorption stages are observed: the zero-adsorption region (pH < 5.5), outstanding increase stage (pH = 5.5 ~ 7), and maximum capacity section (pH > 7). The interesting behaviors might be partially attributed to the surface charges of CNC/CS/PVA-SH and different metal species. Previously, research showed that the heavy metal ions exhibited different valence distribution at pH > 5.0 [43]. The pH value at zero potential point (pH_PZC_) of the synthesized films was about 5.5 (Figure 6a). A positive charge onto the surface of nanofibrous films can be approached at pH < 5.5, which is adversely for M(II) adsorption by the electrostatic repulsion [43]. While the increased adsorption at pH > 5.5 can be associated with the negative surface adsorption. However, when the pH > the buck solution precipitation (pH_BSP_) for metal ions, the hydroxide precipitation will happen.

#### 3.5.3. Kinetics of Adsorption

Adsorption kinetics were used to describe the rate of adsorption reactions. As shown in Figure 7a,b, the metal ion adsorption capacity of the electrospun composite nanofibrous films increased rapidly within 4 h and then decreased. The trend might be related to the higher activity of the multi-dimensional lamellar structure and the higher adsorption activity of the functional groups (–NH_2_ and –SH, with excellent metal chelating ability) of the nanofibrous films in a weak acid. Furthermore, it may also be related to hydrogen bonding (the materials were rich in hydroxyl groups), which increase the capacity to M(II). Under the action of a concentration gradient, the metal ions were rapidly transferred to the outer surface of the films in a short time, and the adsorption process was faster. At the same time, the diffusion of metal ions into lamellar structure of the films was limited, and the adsorption process was slow. The final adsorption sites were occupied by metal ions, and the adsorption was balanced.

**Figure 8:**
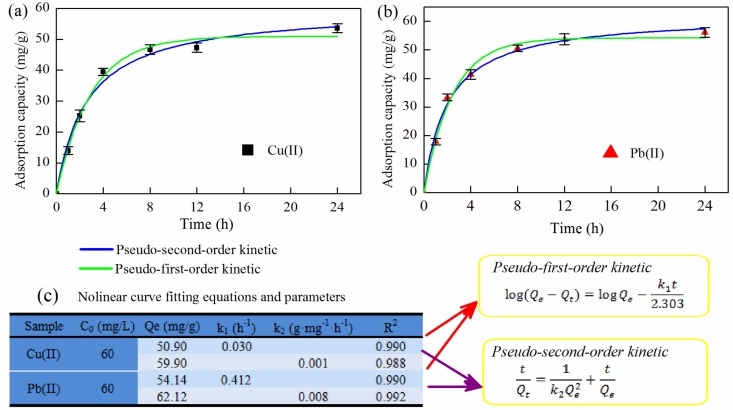
The metal ion, (**a**) Cu(II) and (**b**) Pb(II), adsorption capacity of composite films at different time and kinetics model; (**c**) Fitting parameters of adsorption with kinetic model, *t* is the adsorption time (h), *Q*_t_ is the adsorption capacity at t (mg/g), *Q*_e_ is the equilibrium adsorption capacity (mg/g), and *k*_1_, *k*_2_ are adsorption rate constants.

The adsorption kinetic was used to describe the adsorption of adsorbents to the adsorbate. To better understand the kinetic process, experimental data were fitted by a pseudo-first-order kinetic model and pseudo-second-order kinetic model. The fitting curves and results are shown in Figure 8c. The fitting results showed that the pseudo-second-order kinetic model was consistent with the experimental results. The surface of the composite material was rich in characteristic functional groups (–NH_2_, –OH, and –SH) with metal chelating ability (chemical adsorption) and electrostatic interaction (physical adsorption). The adsorption performance of the modified composite material was higher than that of the unmodified material (Figure 6b). Based on this result, the adsorption behavior was mainly via chemical adsorption. Therefore, the pseudo-second-order kinetic equation can describe the metal ion adsorption performance of the nanofibrous films.

#### 3.5.4. Adsorption Isotherm

**Figure 9:**
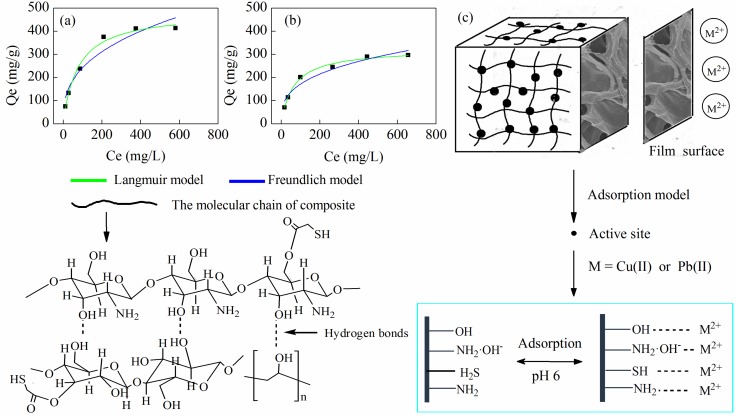
(**a**) and (**b**)Adsorption capacity of the *x*CNC/CS/PVA-SH nanofibrous films for metal ions under different concentration and adsorption isotherms; (**c**) The mechanism of adsorption of M(II) on nanofibrous films.

Adsorption isotherms were used to describe the amount of adsorbent at different adsorbed equilibrium concentrations. The changes in the adsorption capacity of CNC/CS/PVA-SH composite nanofibrous films in different initial heavy metal ions concentrations are shown in Figure 9. The adsorption capacity improved with increasing metal ion concentration. The adsorption capacities of Cu(II) and Pb(II) by the nanofibrous films were 421.61 and 296.30 mg/g, respectively at 25 °C, pH 6.0 and initial heavy metal concentration of 800 mg/g. In the low concentration region, the adsorption capacity increased when the increase of the metal ion concentration was faster. This was because the nanofibrous surface had enough active sites (The activation of –NH_2_ and –SH et al. was higher in solution of pH 6), and the metal ions could be adsorbed better by the material. When the initial concentration of the metal ions was high, the increasing trend of the adsorption capacity was slowed down, and the metal ions were adsorbed and they reached saturation on the surface of the fiber. To a certain extent, this limited the adsorption capacity of the pore surface inside the fiber.

The adsorption capacity of the nanofibrous films with the same initial concentration of metal ions was related not only to the existence of electrostatic attraction and hydrogen bonding between the two, but also to the affinity between the metal ion and the solution. The adsorption process was similar to that of metal ions that were transferred from the solution to the films. The Cu(II) affinity and stability with the solution was higher than those of Pb(II). Therefore, the adsorption performance of the nanofibrous films for Pb(II) was superior to that of Cu(II).

Langmuir and Freundlich models were used to describe the adsorption behavior of metal ions, according to the following Equations (1) and (2):

(1) $$\;Q_{e}=\frac{Q_{\max}bC_{e}}{2a}\;$$

(2) $$Q_{e}=KC_{e}^{1/n}\;$$

In this equation, *Q*_max_ is the maximum adsorption capacity (mg/g); *C*_e_ is the equilibrium adsorption capacity (mg/L); *b* is the Langmuir equation constant (the greater the *b*, the stronger the adsorption); *K* is the Freundlich constant; and, *n* is the nonlinear factor (*n* > 1 means that adsorption is easy to carry out, *n* < 1 means that adsorption is difficult to carry out).

The isothermal equation fitting results are shown in Figure 8. The resulting experimental data are listed in Table 2. The results showed that the adsorption isotherm was described well by Freundlich adsorption isotherm model. The adsorption behavior of composite films for M(II) may be attributed to multi-molecular adsorption. The *n* values of Cu(II) and Pb(II) were 2.75 and 3.06, respectively, and both were greater than 1, indicating that both ions were easily absorbed by composite nanofibrous films under experimental conditions. This was attributed to the rich functional groups and the hydrogen bonding on the fiber surface. Furthermore, the layered-microstructure (Figure 4c) and the ionization state of functional groups on the surface of nanofibrous films also affected the adsorption behavior. Based on the Langmuir model, the maximum adsorption capacities of Cu(II) and Pb(II) ions were estimated at 484.06 and 323.49 mg/g, respectively.

#### 3.5.5. Reusability of the CNC/CS/PVA-SH Composite Nanofibrous Films

**Figure 10:**
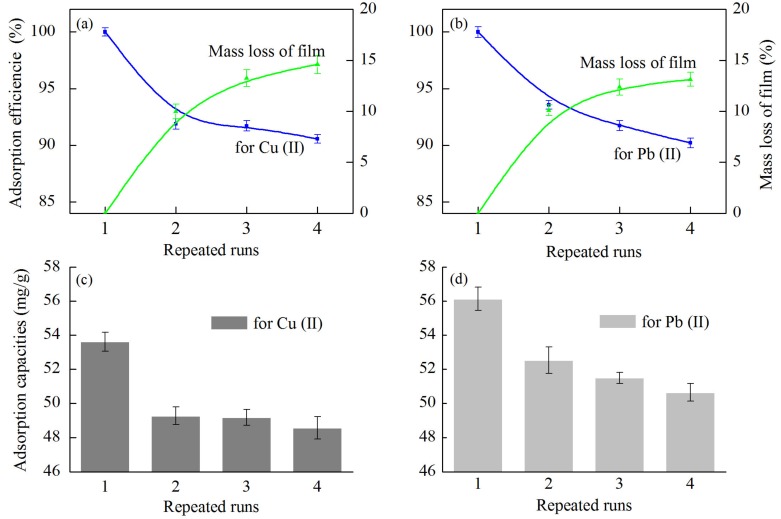
The adsorption efficiency and the stability of the CNC/CS/PVA-SH nanofibrous films for (**a**) Cu(II) and (**b**) Pb(II) ion during repeated runs; The adsorption capacities of (**c**) Cu(II) and (**d**) Pb(II) ion on nanofibrous films.

The main problems associated with desorption and repetitive adsorption of the CNC/CS/PVA-SH composite nanofibrous films are mass loss. The adsorption capacities and the stability during repeated runs are shown in Figure 10. The adsorption capacities of the unit mass of films for M(II) decreased with increasing reuse number, and then stabilized. After 4 repeated runs, the adsorption capacities for Cu(II) and Pb(II) were 48.56 and 50.64 mg/g, respectively, whereby the adsorption efficiencies were 90.58% and 90.21%, respectively, as compared with the first run. This could be explained by the damage of the films, and the residual metal ions present in the material, which decreased the adsorption performance during desorption.

The incorporation of CNC into the CS/PVA mixed matrix can improve the mechanical strength of the materials (Figure 2d), which made materials more tightly bounded due to intramolecular and intermolecular hydrogen bonds, leading to the possible recyclability of the material. With the increase of the number of reabsorption, the mass loss of the films decreased. The mass loss was less than 10% after the four desorption steps. This mass loss was due to the dissolved CS component of the material under the strong acid medium in the desorption process. In addition, both CS and PVA were insoluble in water at room temperature, and the CS and PVA molecules could form a relatively stable hydrogen bonding structure [47], which became more stable with the introduction of CNC. The CS dissolved at pH ≤ 1, and the PVA was insoluble in both acid and alkali. Therefore, it is believable that the adsorbent was relatively stable in aqueous solution.

## 4. Conclusions

The *x*CNC/CS/PVA composite nanofibrous films were fabricated by the electrospinning technique. The surface of the fibers changed from a smooth state to a rough one with increasing CNC content. When 5 wt % of CNC was used, the fibers had regular morphology and uniform diameter distribution. The FTIR results showed that the *x*CNC/CS/PVA nanofibrous films were successfully thiol-functionalized. The SEM analysis showed that the nanofibrous films had a cross-linked porous film structure under weaker acid solution.

The modified films were used for M(II) ion adsorption tests. The best adsorption capacity was obtained for nanofibrous film that was prepared with 5 wt % CNC at pH 6. The adsorption capacity was clearly improved when the initial concentration of M(II) was increased. The adsorption behavior of the nanofibrous films on the M(II) was in accordance with the pseudo-second-order kinetic equation.

From the fitting parameters of adsorption isotherm, the adsorption behavior of the films were agreed with the Freundlich model and may be attributed to multi-molecular adsorption. Based on the Langmuir model, the maximum adsorption capacities of Cu(II) and Pb(II) were estimated at 484.06 and 323.49 mg/g, respectively. The adsorption efficiencies of the films to Cu(II) and Pb(II) after 4 adsorption-desorption cycles were 90.58% and 90.21%, respectively.

## Figures and Tables

**Table 1:** Properties of as-spun solutions.

Solution	Conductivity (μs/cm)	Viscosity (mPa·s)	Surface Tension (mN/m)
0-CNC/CS/PVA	1283	1376	33.7
5-CNC/CS/PVA	1308	1300	36.2
10-CNC/CS/PVA	1315	1304	37.1
20-CNC/CS/PVA	1332	1368	37.7

**Table 2:** Fitting parameters of adsorption isotherm models.

Code	Langmuir Model			Freundlich Model		
Metal Ion	*Q*_max_	*b*	*R* ^2^	*K*	*n*	*R* ^2^
Cu(II)	484.06	0.013	0.986	45.124	2.745	0.911
Pb(II)	323.49	0.015	0.990	37.984	3.058	0.932
